# The future of (non-)surgical ablative therapy in uterine adenomyosis

**DOI:** 10.52054/FVVO.14.4.040

**Published:** 2023-01-27

**Authors:** R.L. De Wilde, R Devassy, H.C. Verhoeven, L.A. Torres-de la Roche

**Affiliations:** University Hospital for Gynecology, PIUS Hospital, Oldenburg, University Medicine Oldenburg, Carl von Ossietzky University, Germany

Uterine adenomyosis (UA) has a significant negative impact on the individual’s quality of life due to pain, abnormal uterine bleeding, and subfertility. The impact on women’s health and sexuality has never been underestimated by physicians, but treatment modalities have been shown to be problematic. Therapies for UA have several insufficiencies to be dealt with. Medical treatment seems successful, but only to a certain degree, and for the duration it is used. Furthermore, it is often counterproductive when used during assisted reproductive therapy (Cope et al., 2020). Even novel drugs such as antiprogestins do not cure but provide remission for a limited period. Surgical treatment on the other hand, although eventually indicated in solitary or localised disease, is difficult in case of diffuse UA, often requiring high expertise and open surgery (Lu et al., 2022) and even then, not always reaching the required level of ‘healing’. This is especially the case when an organ-saving operation is performed in the context of fertility promotion or assisted-reproductive treatment.

As always in medicine, when the existing treatment pathways do not reach curative goals or ensure a high efficacy probability, other options should be evaluated. High-intensity ultrasound (HiFu) ablation, guided by diagnostic ultrasonography, all in the hands of the treating gynaecologist, seems to be a promising tool in UA (Li et al., 2020). Intraoperative assistance of a radiologist is not necessary anymore, as the diagnostic MRI has already been performed preoperatively and the whole of the procedure is monitored by continuous transabdominal ultrasound. During the high- intensity focused energy application, the patient is under sedation. As no general anaesthesia is required, pain evaluation can be carried out by purely asking the patient during the HiFu procedure, thereby avoiding severe complications, due to injury to adjacent organs. Major Asian case studies on large patient series have demonstrated the effectiveness of HiFu, reducing uterine-induced pain, abnormal bleeding and achieving full-term pregnancies, with an acceptable complication rate (Chen et al., 2020). Complications e.g. fever, pain and skin irritation usually disappear during the first 48 hours; major complications such as injury to the nearby organs/structures, e.g., bowel, have a frequency of less than 0.1 %.

Avoiding technically complex major surgery or eventually compromising medical therapy, this alternative treatment modality appears a promising option for diffuse UA (Liu et al., 2022). Following the golden rule of “first do not harm”, the above ablative option requires careful evaluation and consideration to improve the quality of life of women with UA. In 33 case-controlled studies, with more than 100,000 included patients, this method showed comparable results to myomectomy and uterine artery embolisation. The overall satisfaction was high, and a fast recovery allowed early continuation of the socioeconomic life. The genetic influence on the outcome is not yet fully explored; although Afro- American women seem to have equal results, major studies in European are still pending (Torres-de la Roche et al., 2022).

Due to the high importance of the UA, a new Working Group of the European Society for Gynaecological Endoscopy (ESGE)- European Society of Human Reproduction and Embryology (ESHRE) – World Endometriosis Society (WES) has been inaugurated, to give expert recommendations on diagnostics and therapies for UA based on available evidence and experience, described in separate parts. This working group of experts will diligently scrutinise the available evidence before summarising their recommendations. Imaging by MRI and possibilities of transvaginal and transabdominal ultrasound and medical therapies will be evaluated. There will be a special scope on open and laparoscopic surgery, as only a few expert centres are performing adenomyosis surgery on a regular scale. Distinction will be made between solitary or localised disease, and the diffuse form. The non-surgical ablative therapies such as HiFu and uterine artery embolization will also be analysed, highlighting the upcoming importance. It is anticipated that the resulting documents will be published concomitantly both in this journal, FVVO and Human Reproduction Open during the year 2023.
